# Effects of selective β1-adrenoceptor blockade on cardiovascular and renal function and circulating cytokines in ovine hyperdynamic sepsis

**DOI:** 10.1186/s13054-014-0610-1

**Published:** 2014-11-21

**Authors:** Paolo Calzavacca, Yugeesh R Lankadeva, Simon R Bailey, Michael Bailey, Rinaldo Bellomo, Clive N May

**Affiliations:** Florey Institute of Neuroscience and Mental Health, University of Melbourne, 30 Royal Parade, Parkville, VIC 3052 Australia; Department of Intensive Care and Department of Medicine, Austin Health, 145 Studley Road, Heidelberg, VIC 3084 Australia; The Australian and New Zealand Intensive Care Research Centre, 99 Commercial Road, Melbourne, VIC 3004 Australia; Department of Anaesthesia and Intensive Care, AO Melegnano, PO Uboldo, Cernusco sul Naviglio, Italy; Department of Physiology, Monash University, Wellington Road, Clayton, VIC 3800 Australia; Faculty of Veterinary Science, University of Melbourne, Corner Park Drive and Flemington Road, Melbourne, VIC 3052 Australia; Australian and New Zealand Intensive Care Research Centre, Monash University, Wellington Road, Clayton, VIC 3800 Australia

**Keywords:** ᅟ

## Abstract

**Introduction:**

Activation of the sympathetic nervous system has beneficial cardiovascular effects in sepsis, but there is also evidence that sympatholytics have beneficial actions in sepsis. We therefore determined the effect of selective β_1_-adrenoceptor blockade on cardiac and renal function and cytokine release in ovine hyperdynamic sepsis.

**Methods:**

Hyperdynamic sepsis was induced by infusion of live *E. coli* for 24 hours in nine conscious sheep instrumented with flow probes on the pulmonary and left renal artery. Cardiovascular and renal function and levels of plasma cytokines were determined in a control group and during selective β1-adrenoceptor blockade with atenolol (10 mg intravenous bolus then 0.125 mg/kg/h) from 8 to 24 hours of sepsis.

**Results:**

Hyperdynamic sepsis was characterized by hypotension with increases in cardiac output (CO), heart rate (HR) and renal blood flow (RBF), and acute kidney injury. Atenolol caused sustained reductions in HR (*P* <0.001) and CO (*P* <0.001). Despite the lower CO the sepsis-induced fall in mean arterial pressure (MAP) was similar in both groups. The sepsis-induced increase in RBF, decrease in renal function and increase in arterial lactate were unaffected by atenolol. Sepsis increased plasma levels of tumour necrosis factor alpha (TNF-α), interleukin 6 (IL-6) and IL-10. Atenolol caused a further increase in IL-10, but did not affect levels of TNF-α or IL-6.

**Conclusions:**

In sepsis, selective β_1_-adrenoceptor blockade reduced CO, but not MAP. During sepsis, atenolol did not alter the development of acute kidney injury or the levels of pro-inflammatory cytokines, but enhanced the release of IL-10. Atenolol appears safe in sepsis, has no deleterious cardiovascular or renal effects, and has an anti-inflammatory effect.

## Introduction

Widespread activation of the sympathetic nervous system is a well-known phenomenon in sepsis and septic shock [1–3]. An increase in sympathetic nerve activity would be expected to be beneficial in sepsis to maintain arterial pressure and organ perfusion, and indeed treatment with noradrenaline is the first-choice vasopressor to maintain arterial pressure in sepsis [4]. In addition, inotropic treatment with the β-1 adrenoceptor agonist dobutamine has been used to increase cardiac output in sepsis, although a recent study found dobutamine treatment was associated with increased mortality [5] and recent guidelines recommend that dobutamine is used only in selected circumstances [4].

Despite the overall beneficial effects of catecholamines in sepsis, there are indications that a protracted and excessive increase in sympathetic nerve activity during critical illness may become maladaptive and exert adverse effects. For example, an increasing body of evidence suggests that septic myocardial depression and failure can in part be mediated by excessive and prolonged activation of the sympathetic nervous system [6,7]. Indeed, there is evidence in septic shock that inhibiting sympathetic outflow, or blocking the action of catecholamines, may improve survival. Previous studies in sepsis have found contrasting effects depending on whether non-selective or selective β-blockade was used. Non-selective β-blockade with propranolol, started in the first hour of sepsis, improved survival in dogs with endotoxin-induced septic shock [8], and in patients in the late stages of septic shock propranolol improved blood pressure and urine output [9]. In contrast, in mice with sepsis induced by cecal ligation puncture, propranolol caused clinical deterioration and reduced survival [10]. Selective β1-adrenergic blockade with esmolol has been tested in patients with septic shock without a major increase in adverse events [11]. In mice, selective β1-blockade starting two days before treatment with lipopolysaccharide (LPS) or cecal ligation puncture significantly improved survival, although this effect was substantially reduced if β-blockers were not given until 6 h after induction of endotoxemia [12]. In this study, metoprolol reduced plasma levels of interleukin 6 (IL-6) hepatic expression of pro-inflammatory cytokines and cardiac expression of IL-18.

We hypothesised that selective β_1_-adrenoceptor blockade with atenolol would be beneficial in developed hyperdynamic sepsis in conscious sheep by reducing the effects of increased cardiac sympathetic activation and also by decreasing the release of inflammatory cytokines, which in excess may contribute to multiple organ failure. We therefore examined the effects of treatment with the selective β_1_-adrenoceptor antagonist atenolol on arterial pressure and pro- and anti-inflammatory plasma cytokine levels in an ovine model of hyperdynamic, hypotensive sepsis. Since atenolol may also induce cardiovascular decompensation, reduced organ perfusion and organ failure, we measured cardiac output (CO), renal blood flow (RBF) and renal function.

## Materials and methods

### Animal preparation

Experiments were conducted on nine adult Merino ewes (35 ± 1 kg), housed in individual metabolic cages, fed 800 g oaten chaff/day with free access to water. The experimental procedures were approved by the Animal Ethics Committee of the Howard Florey Institute under guidelines laid down by the National Health and Medical Research Council of Australia.

The animals underwent two sterile surgical procedures under general anaesthesia at intervals of two weeks. Anaesthesia was induced with intravenous sodium thiopentone (10 to 15 mg/kg) for intubation with an endotracheal tube (cuffed size 9) and maintained with oxygen/air/isoflurane (end tidal isoflurane 1.5 to 2.0%). Fractional inspired oxygen was altered to maintain SatO_2_ above 97%, and ventilation was controlled to maintain end tidal CO_2_ at approximately 35 mmHg. First, a bilateral carotid arterial loop was created to facilitate arterial cannulation and a transit-time flow probe (Transonics Systems, Ithaca, NY, USA) was placed on the pulmonary artery through a left side 4th intercostal space thoracotomy. During the second procedure, a transit-time flow probe was placed on the left renal artery. The animals were allowed two weeks to recover before the start of experiments. In all operations, animals were treated with intramuscular antibiotics (900 mg, Ilium Propen; procaine penicillin, Troy Laboratories Ptd Ltd, Smithfield, NSW, Australia or Mavlab, Slacks Creek, QLD, Australia) at the start of surgery and then for two days post-operatively. Post-surgical analgesia was maintained with intramuscular injection of flunixin meglumine (1 mg/kg) (Troy Laboratories or Mavlab) at the start of surgery, then 8 and 24 hours post-surgery.

The day before the experiment, a Tygon catheter (Cole-Parmers; Boronia, Australia; ID 1.0 mm, OD 1.5 mm) was inserted 20 cm into the carotid arterial loop to measure arterial pressure and to obtain blood samples. Two polythene catheters were inserted into the superior vena cava via a jugular vein: one for atenolol infusion (Portex™; Smiths Medical International Ltd., Hythe, Kent, UK; ID 1.19 mm, OD 1.7 mm) and one for *Escherichia coli* (*E. coli*) infusion (Portex^™^; Smiths Medical International Ltd.; ID 0.58 mm, OD 0.96 mm). Analog signals (mean arterial pressure (MAP), heart rate (HR), cardiac output (CO) and renal blood flow (RBF) were collected on computer using a customized data-acquisition system (Labview; National Instruments; Austin, TX, USA). Data were recorded at 100 Hz for 10 seconds every minute during experiments.

Blood samples from arterial and central lines, and urine samples, were obtained at the end of 24 hours of baseline, and then at 8, 10, 12 and 24 hours after induction of sepsis for measurement of blood gases, blood lactate (ABL system 625; Radiometer Medical, Copenhagen, Denmark), plasma creatinine (Cr) and urinary Cr, urea, sodium and potassium. Urine was collected in 1-hour lots with an automated urine fraction collector and pooled in 2-hour lots for measurement of creatinine clearance (CreatCl) and fractional excretion of sodium (FENa), potassium (FEK) and urea (FEUN), calculated according to standard formulae: CreatCl = (UCreat x UO)/(PCreat/time(min)) where UCreat is urine creatinine, PCreat is plasma creatinine and UO is urine volume over two hours; FENa, FEK and FEUN = Px/Ux x CrCl x 100 where P is plasma concentration and U is urine concentration of the substance x (sodium, potassium or urea). Oxygen delivery (DO_2_), oxygen consumption (VO) and O_2_ extraction ratio were calculated as per standard formulae: DO_2_ = 10 x CO x arterial O_2_ concentration, VO_2_ = 10 x CO x (arterial - venous O_2_ concentration) and O_2_ extraction ratio =100 x (arterial - venous O_2_ concentration)/(arterial O_2_ concentration).

### Experimental protocol

Following a 24-hour baseline period, sepsis was induced with an intravenous infusion of live *E. coli* (2.8 × 10^9^ colony-forming units, CFU) over fifteen minutes at 8 a.m. followed after 3 hours with a continuous infusion of *E. coli* (1.26 × 10^9^ CFU/h) for 21 hours. At 8 hours after the *E. coli* bolus, when hyperdynamic sepsis had developed and if heart rate had increased by at least 50% compared with baseline, the animals randomly received atenolol (10 mg bolus followed by infusion at 0.125 mg/kg/h in normal saline at 2 ml/h for 16 h) or no treatment.

To determine the effectiveness of cardiac β-1 adrenoceptor blockade with atenolol, the heart rate responses to boluses of the selective β1-agonist isoproterenol (30 ng/kg) were determined. Isoproterenol was given the day before starting the study and at 1, 4, 8 and 16 hours of atenolol after arterial blood samples were collected. At the end of the experiment, infusions of *E. coli* and atenolol were stopped, the animals received intramuscular gentamicin (150 mg), and all catheters were removed. Sheep that survived were crossed over the other arm of the study after at least two weeks.

### Cytokine assays

Arterial blood samples were collected into EDTA tubes on ice at baseline and at 4, 8 and 24 hours of sepsis. Blood was centrifuged at 3,000 g at 4°C for 10 min and plasma was aliquoted and frozen at -80°C. Quantification of interleukin 6 (IL-6) and interleukin 10 (IL-10) levels were performed using in-house enzyme-linked immunosorbent assays (ELISA), as previously described [13]. Tumour necrosis factor alpha (TNF-α) was measured using a commercially available ELISA kit (Kingfisher Biotech Inc., MN, USA). All samples and standards were assayed in duplicate.

### Statistical analysis

Normally distributed data are presented as means ± standard error of means (SEM) and non-normal data as geometric mean (95% confidence interval). Statistical analysis of hemodynamic variables was performed on baseline values (averages of the 24 hours of baseline), and two predefined time intervals of interest during sepsis; the average of the 7th and 8th hour of sepsis (just before atenolol) and the average of the 23rd and 24th hour (at the end of sepsis). For blood tests, all the time points were used for statistical comparisons. Statistical analysis was performed using SAS version 9.2 (SAS Institute Inc., Cary, NC, USA). All variables were assessed for normality and log-transformed where appropriate.

Mixed linear modeling was performed with each sheep treated as a random effect. Main effects were fitted for time and treatment with an interaction between time and treatment used to determine if treatments behaved differently over time. Specific time point comparisons were performed using *post hoc* pairwise *t* tests. To account for multiple comparisons, a reduced *P* value of ≤0.01 was considered to be statistically significant.

## Results

### Cardiovascular responses

**Figure 1:**
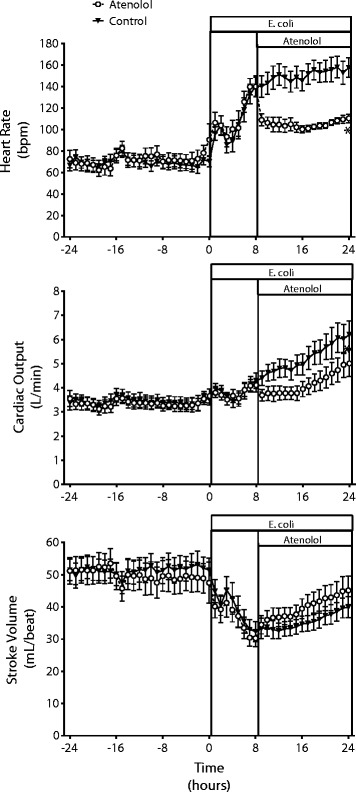
**Cardiac effects of atenolol in ovine hyperdynamic sepsis.** Changes in heart rate, cardiac output and stroke volume in conscious sheep during a 24-hour baseline period and during 24 hours of sepsis induced by intravenous administration of *E. coli* in a control group and a group treated with atenolol from 8 to 24 hours of sepsis. Mean ± SEM, n = 8/group. ^*^ *P* <0.01 for group comparison.

**Figure 2:**
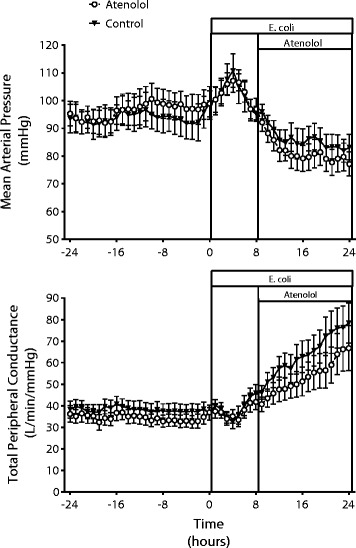
**In ovine hyperdynamic sepsis atenolol did not cause further hypotension.** Mean arterial pressure and total peripheral conductance during a 24-hour baseline period and during 24 hours of sepsis induced by intravenous administration of *E. coli* in a control group and a group treated with atenolol from 8 to 24 hours of sepsis. Mean ± SEM, n = 8/group.

Eight animals per group were studied; one animal in each group died. In the control group, *E. coli* induced hyperdynamic sepsis as shown by the decrease in MAP over 24 hours with increases in CO and HR and a decrease in MAP (Figures 1 and 2, Table 1). The hypotension resulted from peripheral vasodilatation, as shown by the progressive increase in total peripheral conductance (TPC) (Figure 2). In the atenolol group, there were comparable changes in cardiovascular variables to those in the control group at the time atenolol infusion started.

**Table 1:** **Effect of atenolol on hemodynamic variables in ovine hyperdynamic sepsis**

**Cardiovascular variables**	**Gp**	**Baseline**	**Sepsis (8 h)**	**Sepsis (24 h)**
CO (L/min)	A	3.26 (2.80;3.79)	4.01(3.40;4.73)^†^	4.71 (3.99;5.55)^§^*
	C	3.35 2.88;3.90)	4.13(3.50;4.86)^†^	5.86 (4.97;6.90)^§^*
HR (beats/min)	A	68 (59;79)	138 (116;163)^†^	109 (92;129)^§^*
	C	68 (58;78)	133 (113;158)^†^	152 (128;180)^§^*
SV (mL/beat)	A	49.9 ± 4.3	30.4 ± 4.5†	45.0 ± 4.5^§^*
	C	51.3 ± 4.3	34.2 ± 4.5*	41.3 ± 4.5^§^*
MAP (mmHg)	A	95.4 ± 4.9	96.0 ± 5.1	77.2 ± 4.8^§^
	C	93.7 ± 4.9	95.9 ± 5.1	83.2 ± 5.1^§^
TPC (L/min/mmHg)	A	37.0 ± 4.2	44.2 ± 4.6†	69.2 ± 4.6^§^
	C	38.2 ± 4.4	45.0 ± 4.5†	77.4 ± 4.6)^§^
RBF (mL/min)	A	198 (169;238)	268 (220;325)^†^*	286 (236;348)^§^*
	C	212 (178;253)	330 (233;400)^†^*	335 (277;407)^§^*
RVC (mL/min/mmHg)	A	2.11 (1.78;2.50)	2.81(2.31;3.41)^†^	3.75 (3.09;4.55)^§^
	C	2.32 (1.99;2.75)	3.48(2.88;4.21)^†^	3.51 (2.93;4.2)^§^

Measurements were taken during a 24-hour baseline, at 8 hours of sepsis (mean of 7^th^ and 8^th^ hour) and at 24 hours of sepsis (mean of 23^rd^ and 24^th^ hour) in a control group (C) and a group treated with atenolol from 8 to 24 hours of sepsis (A) (n = 8/group). Values are mean ± SEM for normally distributed or geometric mean (95% confidence interval) for non-normally distributed variables. ^†^ *P* ≤0.01 for baseline vs. 8 hours of sepsis; ^§^ *P* ≤0.01 for 8 hours of sepsis vs. 24 hours of sepsis; ^*^ *P* ≤0.01 for Aten vs. Con. CO: cardiac output; HR: heart rate; SV: stroke volume; MAP: mean arterial pressure; TPC: total peripheral conductance; RBF: renal blood flow; RVC: renal vascular conductance.

Treatment of septic sheep with atenolol, caused an immediate decrease in HR from 138 (116,163) beats/min, which was maintained at this lower level until the end of the 16-hour infusion (109(92,129) beats/min) (Figure 1, Table 1). Atenolol also reduced CO within 1 hour and although HR remained reduced there was a slow increase in CO due to a progressive increase in stroke volume (SV) during the septic period (Figure 1, Table 1). Despite the increase in SV in the atenolol group, CO remained below the level in the control group throughout the septic period. Although atenolol decreased CO, the level of MAP during sepsis was similar in the atenolol and control groups (77.2 ± 4.8 vs. 83.2 ± 5.1 mmHg respectively, *P* = 0.86) (Figure 2). This was due to the smaller increase in TPC in the atenolol group, which although not significant was sufficient to prevent a greater level of hypotension during atenolol treatment. Effective β_1_-adrenergic blockade by atenolol was confirmed by demonstrating that the tachycardic response to a bolus of isoproterenol was blocked. Isoproterenol (30 ng/kg) given before atenolol increased HR by 43 ± 13 beats/min, while after atenolol there was no significant change at any time point (maximum increase 3 ± 1 beats/min).

During the development of sepsis, there were no significant changes in partial pressure of oxygen (pO_2_), DO_2_, oxygen volume (VO_2_) or O_2_ extraction ratio (Table 2). By 24 hours of sepsis, DO_2_ had significantly increased in the control (*P* = 0.001) not the atenolol group (*P* = 0.016), but there were no differences in DO_2_ between groups at any time point (see Table 2). Similarly, during sepsis, compared with baseline, VO_2_ and O_2_ extraction ratio were not changed, except for O_2_ extraction ratio at 24 hours of sepsis that was significantly decreased in the control group (*P* = 0.007). No differences were observed between groups for VO_2_ and O_2_ extraction ratio. There was a similar threefold increase in arterial lactate in both groups (Table 2).

**Table 2:** **Effect of atenolol on oxygen variables in ovine hyperdynamic sepsis**

**Biochemical variables**	**Gp**	**Baseline**	**Sepsis (8 h)**	**Sepsis (10 h)**	**Sepsis (12 h)**	**Sepsis (24 h)**
pO_2_ (mmHg)	A	90 ± 2	84 ± 2	86 ± 3	86 ± 3	77 ± 2
	C	86 ± 3	78 ± 4	78 ± 4	79 ± 5	75 ± 6
SvcO_2_ (%)	A	68.2 ± 2.9	72.3 ± 3.3	70.0 ± 2.2	69.4 ± 3.0	72.5 ± 3.9
	C	54.6 ± 4.6	71.2 ± 1.7	67.3 ± 6.9	74.8 ± 3.6	72.9 ± 1.2
DO_2_ (mL O_2_/min)	A	463 (400;536)	561 (485;650)	496 (427;577)	513 (441;597)	605 (522;700)
	C	489 (422;566)	590 (502;692)	597 (508;702)	619 (527;728)	706 (610;818)^†^
VO_2_ (mL O_2_/min)	A	167 ± 17	168 ± 17	136 ± 17	145 ± 17	142 ± 17
	C	146 ± 17	144 ± 17	154 ± 19	154 ± 19	143 ± 17
O_2_ E (%)	A	31.4 (24.2;40.3)	27.9 (21.2;36.8)	27.1 (20.7;35.5)	27.3 (20.9; 35.7)	19.3 (15;24.8)
	C	33.2 (25.9;42.7)	24.1 (18.7;30.9)	24.6 (18.4;32.8)	23.8 (17.8;31.8)	22.4 (17.4;28.8)^†^
Lactate (mmol/L)	A	0.37 (0.24;0.56)	1.20 (0.79;1.83)^†^	1.61 (1.05;2.49)^†^	1.57 (1.02;2.41)†	1.31 (0.86;1.99)^†^
	C	0.39 (0.26;0.59)	1.37 (0.9;2.08)^†^	1.54 (1.00;2.37)^†^	1.55 (1.01;2.39)†	1.34 (0.88;2.04)^†^

Oxygen variables and arterial lactate during 24 hours of baseline and at 8, 10, 12 and 24 hours of sepsis in the control group (C) and the group treated with atenolol from 8 to 24 h of sepsis (n = 8/group). Values are mean ± SEM or geometric mean (95% confidence interval) for normally and non-normally distributed variables, respectively. ^†^ *P* ≤0.01 for time change vs. baseline (within group comparison). pO_2_: partial pressure of oxygen; SvcO_2_: superior vena cava O_2_ saturation; DO_2_: O_2_ delivery; VO_2_: O_2_ consumption; O_2_E: oxygen extraction ratio.

### Renal responses

**Figure 3:**
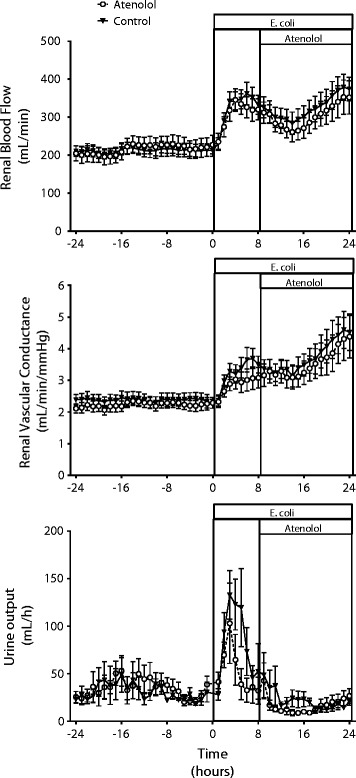
**Renal effects of atenolol in ovine sepsis.** Changes in renal hemodynamics and urine output in conscious sheep during a 24-hour baseline period and during 24 hours of sepsis induced by intravenous administration of *E. coli* in a control group and a group treated with atenolol from 8 to 24 hours of sepsis. Mean ± SEM, n = 8/group.

During sepsis there was a large increase in RBF due to intense renal vasodilatation as shown by the almost doubling in renal vascular conductance (RVC) by 24 hours of sepsis (Figure 3). Sepsis caused an initial transient diuresis followed by oliguria, and the development of acute kidney injury was demonstrated by the increase in plasma creatinine and decrease in CreatCl (Figure 3, Table 3).

**Table 3:** **Renal effects of atenolol in ovine sepsis**

**Renal variables**	**Gp**	**Baseline**	**Sepsis (8 h)**	**Sepsis (10 h)**	**Sepsis (12 h)**	**Sepsis (24 h)**
Urine (mL/h)	A	24.2 (17.3;33.7)	19.7 (12.2;31.9)	15.8 (9.8;25.6)	10.6 (6.5;17.3)	17.9 (11.0;29.0)
	C	22.2 (15.9;31.0)	30.3 (18.5;49.5)	21.2 (13.1;34.4)	14.2 (8.8;23.0)	14.7 (9.1;23.9)
P-creat (μmol/L)	A	73 (60;88)	108 (90; 130)^†^	123 (100;150)^†^	138 (113;168)^†^	158 (131;191)^†^
	C	62 (51;75)	84 (70;101)	90 (73;109)	97 (79;118)^†^	132 (110;160)^†^
CreatCl (mL/min)	A	58.5 ± 7.1	30.9 ± 7.0^†^	26.5 ± 7.7^†^	16.4 ± 7.7^†^	36 ± 7.1
	C	66.1 ± 7.1	43.4 ± 7.0^†^	45.3 ± 7.7	37.5 ± 7.7^†^	39.7 ± 7.1^†^
FENa (%)	A	0.21 (0.11;0.43)	0.42 (0.21;0.85)	0.32 (0.16;061)	0.51 (0.26;0.98)	0.13 (0.07:0.26)
	C	0.18 (0.09;0.39)	0.48 (0.24;0.96)	0.26 (0.14;0.50)	0.20 (0.10;0.38)	0.08 (0.04;0.15)
FEK (%)	A	29.7 ± 3.9	25.7 ± 3.9	26.3 ± 4.9	27.9 ± 4.9	27.2 ± 3.9
	C	23.7 ± 3.9	25 ± 3.9	20.2 ± 4.9	18.0 ± 4.9	25.9 ± 3.9
FEUN (%)	A	48.2 ± 7.6	35.9 ± 7.1	32.3 ± 7.2	31.2 ± 7.2	17.5 ± 7.1^†^
	C	47.9 ± 7.1	42.7 ± 7.1	31.5 ± 7.2	29.4 ± 7.2	22.0 ± 7.1^†^

Renal function parameters during 24 hours of baseline and at 8, 10, 12 and 24 hours of sepsis in the control group (C) and the group treated with atenolol from 8 to 24 hours of sepsis (n = 8/group).Values are mean ± SEM or geometric mean (95% confidence interval) for normally and not-normally distributed variables, respectively. ^†^ *P* ≤0.01 for time change vs. baseline (within group comparison). P-creat: plasma creatinine; CreatCl: creatinine clearance; FENa: fractional excretion of sodium; FEK: fraction excretion of potassium; FEUN: fractional excretion of urea nitrogen. BP values: mean of 24-hour baseline period.

Atenolol had only minor effects on the sepsis-induced changes in renal hemodynamics and renal function. Atenolol did not affect RBF or RVC during the intervention period (*P* = 0.925 and 0.556, respectively). The initial polyuric response following *E. coli* was more pronounced in the control group, but oliguria of a similar magnitude developed in both groups by the end of sepsis (Figure 3, Table 3). Plasma creatinine increased more in the atenolol than the control group at 2 and 4 hours after starting atenolol (Table 3, *P* = 0.003 and <0.001, respectively), and although it tended to be higher after 16 hours of atenolol, at 24 hours of sepsis, this difference was not significant (*P* = 0.072). There were no significant differences in CreatCl, FENa, FEK or FEUN between the two groups at any time point (Table 3).

### Plasma cytokines

**Figure 4:**
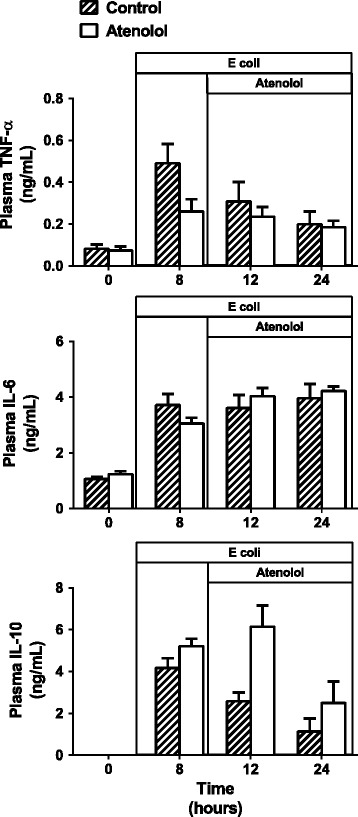
**Effects of atenolol on plasma cytokine levels in ovine sepsis.** Plasma cytokine levels in conscious sheep during a 24-hour baseline period and during 24 hours of sepsis induced by intravenous administration of *E. coli* in a control group and a group treated with atenolol from 8 to 24 hours of sepsis. Mean ± SEM, n = 8/group.

The plasma levels of all cytokines measured were low during the control period and increased during sepsis (Figure 4). Plasma TNF-α was significantly increased in the control group at 8 hours of sepsis (from 0.1 (0.05; 0.18) to 0.41(0.22; 0.74) ng/mL (*P* <0.01), followed by a fall towards baseline levels by 24 hours of sepsis, and there was no significant difference between the control and atenolol groups over time (Figure 4). Plasma IL-6 was significantly increased in the control group by 8 hours of sepsis (from 1.04 (0.86; 1.26) to 3.57 (2.96; 4.30) ng/mL) (*P* <0.001) and remained elevated at a similar level until 24 hours of sepsis. The sepsis-induced increase in IL-6 was not changed by treatment with atenolol. Plasma IL-10 also significantly increased by 8 hours of sepsis from undetectable levels to 5.11 (3.84; 6.82) ng/mL (*P* <0.001), but had returned to control levels by 24 hours of sepsis. Treatment with atenolol significantly attenuated the decline in IL-10 compared with control sheep (*P* <0.01) (Figure 4).

## Discussion

The main findings of this study are that in an ovine model of hyperdynamic sepsis selective β1-adrenoceptor blockade with atenolol reduced HR and CO, but increased SV. Despite the greater fall in CO during atenolol treatment, the level of hypotension that developed during sepsis was similar to that in the control group. Atenolol did not alter the sepsis-induced renal vasodilatation and increase in RBF and by the end of the atenolol infusion there was a similar level of acute kidney injury. Atenolol did not decrease oxygen extraction or increase arterial lactate levels during sepsis. During sepsis plasma levels of TNF-α, IL-6 and IL-10 increased; atenolol further increased the level of the anti-inflammatory cytokine IL-10, but had no effect on the pro-inflammatory cytokines TNF-α and IL-6.

There is increasing interest in the effects of sympatholytic drugs in sepsis. Recent human trials have shown that esmolol treatment in patients with pneumonia and in septic shock was not associated with major adverse events despite a reduction in CO and DO_2_ [11,14]. In addition, patients on chronic β-blocker therapy admitted to intensive care with sepsis had reduced mortality [15]; such findings may promote the more widespread use of these agents. Similarly, in some experimental studies, β-blockers improved survival in sepsis [8,12], but it remains unclear why these drugs, which would be expected to be detrimental in a hypotensive disease, had a beneficial action and reduced mortality. It may depend on the reduction in tachycardia leading to improved cardiac function [16] or a reduction in the level of inflammatory cytokines [12,17], since these molecules can themselves cause organ damage and even death, independently of the underlying infection [18].

In the present study the dose of atenolol used completely blocked cardiac β1-adrenoceptors, as judged by the inhibition of the tachycardic effect of isoproterenol. Interestingly, this dose of atenolol did not reduce HR to control levels, indicating that the sepsis-induced tachycardia was not completely dependent on increased cardiac sympathetic activation. This is in agreement with a previous study in which we examined the arterial baroreflex control of HR in sepsis and concluded that, in addition to sympathetic activation, a reduction in cardiac vagal activity and/or a direct effect of cytokines on the heart accounted for the tachycardia in sepsis [2]. Although atenolol treatment increased SV, this was insufficient to offset the effect of the reduction in HR and throughout the infusion CO remained lower in the atenolol group. This reduction in CO did not result in a greater degree of hypotension during sepsis because there tended to be less peripheral vasodilatation in the atenolol-treated group. The mechanism for this is unclear but is most likely to be a peripheral vascular effect because atenolol is hydrophilic and does not cross the blood-brain barrier. Indeed there is evidence that atenolol causes peripheral vasoconstriction, which has been proposed to be either by a reflex increase in sympathetic activity or blockade of prejunctional β-adrenoceptors [19].

A similar maintenance of blood pressure, with a fall in CO and increase in peripheral resistance, has been reported with propranolol treatment in patients with hyperdynamic septic shock [9]. The finding that requirement for norepinephrine was reduced in patients with septic shock treated with the β-_1_ adrenoceptor blocker, esmolol [20], suggests that treatment with β-blockers may improve vascular reactivity in sepsis. The present findings indicate that in sheep with hyperdynamic sepsis arterial pressure is maintained in the presence of selective cardiac sympathetic denervation, whereas we have recently shown that selective renal denervation resulted in a greater degree of hypotension [21].

As we have previously shown, hyperdynamic sepsis was accompanied by renal vasodilatation, an increase in RBF and acute kidney failure [22,23]. In septic sheep, atenolol had minor effects on renal hemodynamics and renal function despite causing a decrease in CO, probably because atenolol did not induce a greater degree of hypotension. Critically, renal function in the atenolol group, estimated by changes in creatinine clearance, plasma creatinine, urine output, FENa, FEK and FEUN, was similar to that in the control group. In addition, indices of tissue perfusion, oxygen consumption and oxygen extraction, were similar in the atenolol and control groups, as was the increase in blood lactate, indicating similar levels of disease severity in both groups.

Our findings of simultaneous increases in both pro-inflammatory and opposing anti-inflammatory cytokines in this ovine model of sepsis are in accord with findings in septic patients [24–26]. Although the levels of TNF-α and IL-10 declined towards normal from 8 to 24 hours of sepsis, the increase in IL-6 was maintained throughout the septic period indicating a maintained hyper-inflammatory phase. Treatment with atenolol did not alter the levels of TNF-α or IL-6, but increased IL-10, indicating no effect of β1-adrenoceptor blockade on the pro-inflammatory response, but an enhancement of the anti-inflammatory response. Currently the mechanism by which atenolol increased IL-10 in ovine sepsis is unclear since in isolated macrophages β-agonists stimulate IL-10 release [27,28], suggesting that in the whole animal other mechanisms override this effect.

These findings vary from those in murine sepsis where β1-blockade with metoprolol, started two days before sepsis, reduced plasma IL-6 and hepatic expression of key inflammatory genes and reduced mortality, although if treatment was given 6 hours after endotoxaemia there was no significant improvement in survival [12]. The β1-adrenoceptor blocker landiolol reduced both TNF-α and IL-6 and protected against acute lung injury and cardiac dysfunction in LPS-treated rats [29]. In rats, following cecal ligation puncture, esmolol reduced plasma TNF-α, but not IL-1β, and increased myocardial β1-receptor expression [16]. These differences from the present study are probably in part species related considering the vastly different sensitivity to LPS across species, with humans and sheep having a 500 to 1,000 times greater sensitivity than rats and mice [30]. In addition, the different genomic responses to inflammatory stimuli in mice and humans have also questioned the use of murine models of sepsis [31].

Our study has both strengths and limitations. The major strength was that it reproduced severe septic acute kidney injury in a large conscious mammal and described the effects of selective β_1_-adrenoceptor blockade, started at a clinically relevant time when signs of sepsis were present, on cardiovascular function, renal function and cytokine levels. The indirect estimation of glomerular filtration rate (GFR) using creatinine clearance is of limited accuracy, but this method is widely used and clinically relevant. Furthermore, its changes were concordant with changes in plasma creatinine. We only investigated the effects of selective β1-adrenocetor blockade so we are not able to distinguish the extent to which the findings are due to β_1_-antagonism or selective stimulation of β_2_-adrenoceptors. As in all experimental animal studies, the relevance of the findings to the clinical situation is unclear, but as most human sepsis is hyperdynamic in nature it is likely that this model has clinical relevance.

## Conclusions

In experimental hyperdynamic sepsis treatment with atenolol, started at a clinically relevant time when sepsis had developed, did not lead to greater hypotension even though CO was reduced. Renal function and indices of tissue perfusion were not adversely affected by atenolol and disease severity appeared similar in the control and treated groups, indicating the safety of the treatment. Atenolol did not influence the plasma levels of the pro-inflammatory cytokines TNF-α and IL-6 but it induced a greater increase in the anti-inflammatory cytokine IL-10. These findings indicate in hyperdynamic sepsis, with a relatively small decrease in arterial pressure and a large increase in CO, that treatment with atenolol had no detrimental cardiovascular or renal effects and had an anti-inflammatory effect. Further studies are required to determine if atenolol is safe in septic shock with circulatory failure.

## Key messages

- In an ovine model of hyperdynamic sepsis atenolol reduced heart rate and cardiac output
- In sepsis atenolol did not cause a greater degree of hypotension.
- Atenolol did not reduce renal perfusion or worsen the development of septic acute renal failure.
- Atenolol did not reduce the sepsis-induced increases in the inflammatory cytokines TNF-α or IL-6 but increased the level of the anti-inflammatory cytokine IL-10.

## Abbreviations

CO: cardiac output
Cr: plasma creatinine
CreatCl: creatinine clearance
DO_2_: oxygen delivery
ELISA: enzyme-linked immunosorbent assay
FENa: fractional excretion of sodium
FEK: fractional excretion of potassium
FEUN: fractional excretion of urea
GFR: glomerular filtration rate
HR: heart rate
IL: interleukin
LPS: lipopolysaccharide
MAP: mean arterial pressure
pO_2_: partial pressure of oxygen
RBF: renal blood flow
RVC: renal vascular conductance
SEM: standard error of the mean
SV: stroke volume
TPC: total peripheral conductance
TNF-α: tumour necrosis factor alpha
VO_2_: oxygen consumption
